# Cost-effectiveness of cervical cancer screening and HPV vaccination: a Markov model from the healthcare payer perspective

**DOI:** 10.3389/fpubh.2025.1508623

**Published:** 2025-10-21

**Authors:** Weixia Liu, Li Jing, Xiaojun Huang, Hongxin Huang, Kuan Lie Jiang, Wei Lu

**Affiliations:** ^1^Hainan Medical University, HaiKou, Hainan, China; ^2^Hainan Open University, HaiKou, Hainan, China

**Keywords:** cervical cancer, cost-effectiveness analysis, HPV vaccine, screening strategies, Markov model

## Abstract

**Background:**

Substantial progress has been made in cervical cancer screening and HPV vaccination in China. However, evidence on the cost-effectiveness of these interventions remains scarce, particularly for combined vaccination and screening strategies at the provincial level. To address this gap, we evaluated the cost-effectiveness of alternative cervical cancer prevention strategies in a southern province of China from the healthcare payer perspective.

**Methods:**

A Markov model was constructed to simulate a cohort of 100,000 females beginning at age 9 and followed until death (up to 100 years). The model compared the outcomes of bivalent, quadrivalent, and 9-valent HPV vaccines combined with two screening methods: TCT and HPV testing. Analyses were conducted from the healthcare payer perspective, considering only direct medical costs. The primary outcome was quality-adjusted life years (QALYs), discounted at 3% annually. Herd immunity effects were not incorporated. Model calibration relied on data from the China Health Statistics Yearbook, and sensitivity analyses assessed parameter uncertainty. Reporting followed the CHEERS 2024 guidelines.

**Results:**

Thirteen strategies were evaluated, including no intervention, screening alone, and combinations of screening with the three HPV vaccines. The combination of HPV testing and the 9-valent vaccine was the most cost-effective, with an incremental cost-effectiveness ratio (ICER) of ¥139.58 per QALY, well below the willingness-to-pay threshold. By contrast, TCT combined with the 9-valent vaccine yielded the highest ICER at ¥193,240.60 per QALY, exceeding the threshold. Sensitivity analyses showed ICER estimates were most influenced by screening coverage, vaccination uptake, test sensitivity, and the discount rate.

**Conclusion:**

Within the current resource and policy context, combining HPV testing with the 9-valent vaccine provides the highest economic value. This strategy offers evidence to guide future cervical cancer prevention policies in southern China.

## 1 Introduction

Persistent infection with human papillomavirus (HPV) is the leading cause of cervical cancer and several other malignancies, including cancers of the anus, vulva, vagina, penis, and head and neck. Cervical cancer is the fourth most common cancer among women worldwide, imposing a substantial global health burden. In 2020, China reported approximately 110,000 new cases and 60,000 deaths from cervical cancer, accounting for 18% of global incidence and 17% of mortality, corresponding to approximately 20.2% of the global disease burden (1, 2).

In 2018, the World Health Organization (WHO) launched the Global Initiative to Eliminate Cervical Cancer, followed in 2020 by the Global Strategy to Accelerate Elimination. This strategy is built on three pillars—vaccination, screening, and treatment—with the target of reducing cervical cancer incidence to fewer than four cases per 100,000 women and ultimately achieving elimination (3, 4).

In China, prevention efforts primarily focus on primary and secondary measures. Primary prevention emphasizes HPV vaccination among girls of appropriate age, while secondary prevention includes screening, early detection, and timely treatment in target populations (5). Although vaccines can substantially reduce cervical cancer risk, they do not provide lifelong immunity. Timely screening is essential to prevent disease progression and reduce incidence and mortality. A combined approach of vaccination and screening is widely regarded as the most effective strategy, and many countries have evaluated the cost-effectiveness of this approach (6, 7). Evidence suggests that large-scale, organized screening combined with bivalent HPV vaccination can significantly reduce the incidence and mortality of cervical cancer and its precursors, while also achieving substantial herd immunity benefits (8–10).

However, economic evaluations of all three available HPV vaccines in combination with current large-scale screening strategies in China remain limited. Therefore, this study aimed to assess the cost-effectiveness of HPV vaccination and screening strategies in a southern province of China from the healthcare payer perspective, with the aim of providing evidence to inform policy-making and program implementation.

## 2 Materials and methods

### 2.1 Intervention strategies

A Markov model was developed using TreeAge Pro to simulate the natural progression of cervical cancer in a cohort of 100,000 females starting at age 9 and followed until death, with a maximum age of 100 years. The model comprised 12 mutually exclusive health states, representing the full disease spectrum from HPV infection through CIN1/2/3 lesions to cervical cancer and death. A one-year cycle length and lifetime horizon were applied.

**Figure 1:**
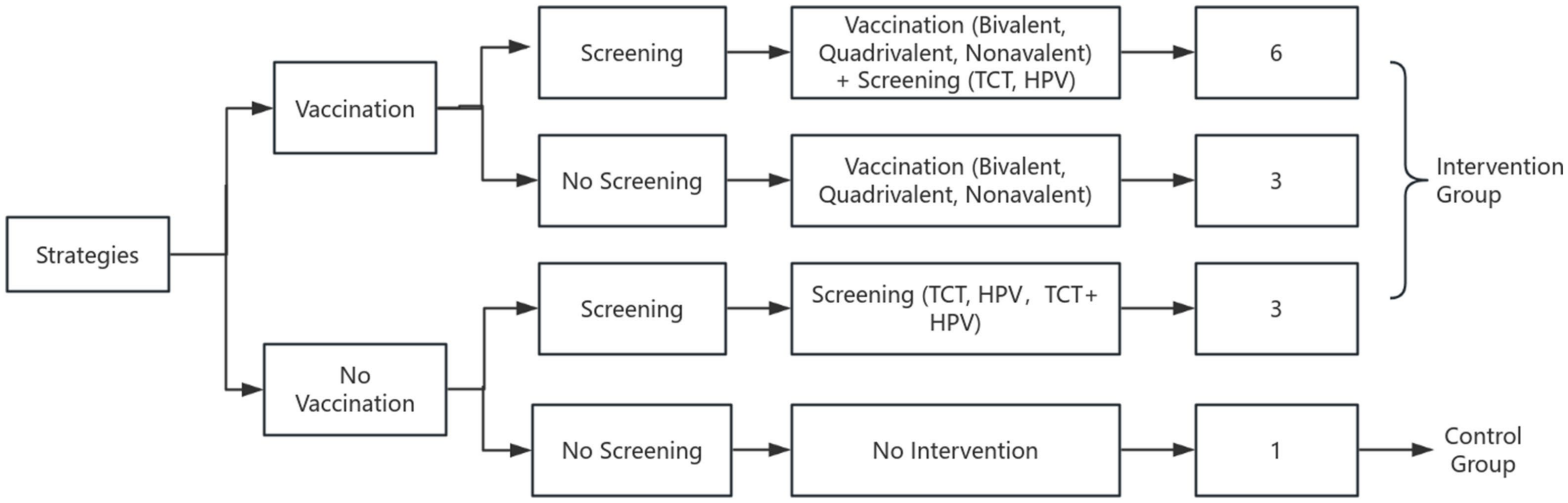
Logical flowchart of cervical cancer prevention strategies.

Three HPV vaccination strategies were evaluated: bivalent, quadrivalent, and 9-valent vaccines. Vaccination was assumed to be completed at age 9 and administered exclusively to females. Details of the intervention strategies and model framework are shown in Figure 1.

Based on a national survey reporting that approximately 70% of women aged 27–45 in China are willing to receive HPV vaccination, coverage rate was set at 70% (11). Screening strategies included TCT (ThinPrep Cytologic Test) every 3 years and HPV DNA testing every 5 years. Considering evidence that around 80% of Chinese women are willing to participate in cervical cancer screening, the screening coverage rate was set at 80% (12). In total 13 intervention strategies were evaluated, including a no-intervention control group. Figure 1 presents the logical flowchart of the cervical cancer prevention strategies. Model cycles ended when cervical cancer death, death from other causes, or cohort dropout occurred, at which point individual exited the simulation. From the healthcare payer perspective, herd immunity effects and male vaccination were excluded.

The Markov model assumed that patients transition between different disease states based on specific transition probabilities (13).

In the literature, these probabilities are typically derived from incidence rates, which are converted using standard formulas.

**Figure 2:**
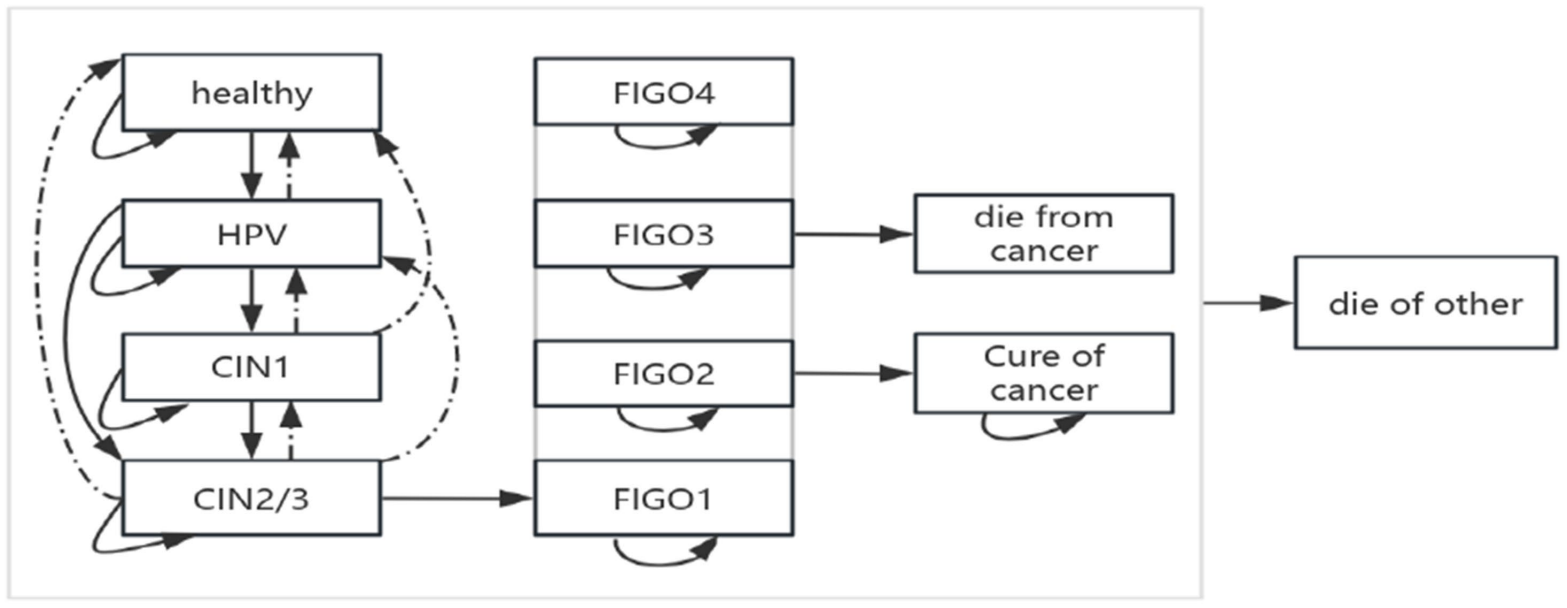
The structure of the modelled natural history.

In this study, transition probabilities represented the likelihood that individuals in a given state would progress, remain stable, or regress in the next cycle, thereby reflecting the natural history of the disease (14). The modelled structure of disease progression is shown in Figure 2.

### 2.2 Input parameters

#### 2.2.1 Epidemiological parameters

The model included 12 health states representing the natural history of cervical cancer, ranging from susceptibility to advanced disease: Healthy, HPV Infection, Cervical Intraepithelial Neoplasia Grade I (CIN1), Cervical Intraepithelial Neoplasia Grade II (CIN2), Cervical Intraepithelial Neoplasia Grade III (CIN3), and FIGO stages I–IV cervical cancer. Additional states include: death due to cervical cancer (die from cancer), death due to other causes (die of other), and exit from the cohort (out). Transitions between different health states are facilitated by delineating transition pathways and assigning probabilities, with specific transition probability parameters presented in Table 1. The utility values for different states are provided in Table 2.

**Table 1:** Probability of metastasis between states of cervical cancer.

Variable	Age	Value	Range	Distribution type	Reference
Health to HPV	10-	0.0000		Beta	Zhao F H et al. (22)
	15-	0.0000			
	20-	0.1728			
	25-	0.1363			
	30-	0.1429			
	35-	0.1818			
	40-	0.188			
	45-	0.1962			
	50-	0.1648			
HPV back Health	15-	0.600			Mo X et al. (23)
	20-	0.600			
	25-	0.350			
	30-	0.300			
HPV to CIN 1		0.0717	0.0527 ~ 0.1121		Canfell K et al. (24–26)
HPV to CIN 2		0.0115	0.0034 ~ 0.0234		
HPV to CIN 3		0.0115	0.0034 ~ 0.0234		
CIN 1 back Health		0.708	0.6077 ~ 0.7933		
CIN 1 back HPV		0.0150	0.2623 ~ 0.4372		
CIN 1 to CIN 2		0.2240	0.1608 ~ 0.2972		
CIN 1 to CIN 3		0.0464	0.0098 ~ 0.1297		
CIN 2 to CIN 3		0.3498	0.2623 ~ 0.4372		
CIN 2/3 to FIGO 1		0.1019	0.0764 ~ 0.1274		
CIN 2/3 back CIN 1		0.0135	±25%		
CIN 2/3 back HPV		0.1901	±25%		
CIN 2/3 back Health		0.0690	0.0564 ~ 0.0774		Jie J Q (27)
FIGO 1 to FIGO 2		0.4377	±25%		Canfell K et al. (24–26)
FIGO 2 to FIGO 3		0.5358	±25%		
FIGO 3 to FIGO 4		0.6838	±25%		
FIGO 1 to death from cancer		0.025	±25%		
FIGO 2 to death from cancer		0.078	±25%		
FIGO 3 to death from cancer		0.144	±25%		
FIGO4 to death from cancer		0.444	±25%		
death from cancer	30-	0.2381			Zhang Y (28)
	35-	0.2381			
	40-	0.2675			
	45-	0.2674			
	50-	0.332			
	55-	0.3331			

**Table 2:** Utility values for each state of cervical cancer.

State of health	Utility value	Distribution type	Reference
Health	1	Beta	Model Assumptions Based on Expert Recommendations and Literature References
HPV	1		
CIN 1	1		
CIN2	0.8760		Li M (29)
CIN3	0.8060		Woodhall SC (30)
FIGO 1	0.697		
FIGO 2	0.641		
FIGO 3	0.636		
FIGO 4	0.590		
Cancer cured	0.84		Mo X (31)

#### 2.2.2 Effectiveness

Effectiveness in this study was measured in terms of QALYs. Most utility inputs were derived from previously published cost-effectiveness studies based on Chinese populations. However, due to the lack of stage-specific domestic data on cervical cancer utilities, estimates for different cancer stages were adopted from a European cost-utility analysis.

The European estimates were selected based on the methodological rigor of the original study, relevance to clinical staging, and consistency with international literature. While these values may not fully reflect the health preferences of the Chinese population, they provide a reasonable proxy in the absence of local data. A complete list of utility parameters is provided in Table 2.

#### 2.2.3 Medical and vaccine costs

Comprehensive cost data were collected for two screening methods and three vaccines from the healthcare payer perspective, based on real-world conditions in a southern province of China. Costs were categorized as direct medical, direct non-medical, and indirect.

Identification and measurement of costs: Direct medical costs were obtained from inpatient medical records at a tertiary hospital in the province and included registration fees, administrative fees, and healthcare personnel time. Registration fees were measured using local healthcare service prices, while personnel time was valued according to the 2023 average wages of public sector employees reported by the National Bureau of Statistics of China. Direct non-medical costs (e.g., transportation and food expenses for patients and family members) were measured using patient-reported expenditure data and valued at prevailing market prices. Indirect costs referred to productivity losses from reduced work hours due to illness and premature mortality, estimated using the human capital approach and valued at 2023 average wage levels.

Valuation of costs: all costs were expressed in 2023 Chinese Yuan (CNY). Historical prices were adjusted using the local Consumer Price Index where necessary. Future costs and benefits were discounted at an annual rate of 3%, consistent with previous cost-effectiveness studies in China.

Exclusion of costs: informal caregiving, intangible costs (e.g., psychological burden), and long-term social support expenditures were excluded due to a lack of reliable data.

Based on this analysis, the estimated average economic burden per case was: 2,030.78 CNY for HPV infection, 7,176.38 CNY for CIN I, 9,627.23 CNY for CIN II, 9,931.17 CNY for CIN III, and 19,307.48 CNY for cervical cancer. Ranges of screening and vaccination costs are summarized in Table 3.

**Table 3:** Summary of cost parameters for vaccination and screening.

Cost category	Specific categories	Costs (CNY)	Minimum	Maximum	Reference
Vaccinations	2-valent vaccine - three doses + administration fee	1,490	745.00	2,235	Zhang Q (32)
	4-valent vaccine - three doses + administration fee	2,979	2,899	3,088	
	9-valent vaccine - three doses + administration fee	4,499	4035.00	5380.00	
Screening	HPV testing	66.40	64.70	70.34	2023 screening program in a southern province
	TCT screening	66	65.70	69.28	
	HPV + TCT	132.4	130.70	133.30	

In the Markov model, costs were assigned to both health states and interventions. Each health state (e.g., HPV infection, CIN, cervical cancer stages, post-treatment) was associated with annual management costs. Screening and vaccination costs were applied at the time of intervention, while false-positive results incurred additional diagnostic costs. Costs were assumed constant across age groups, consistent with prior cost-effectiveness analyses in China.

#### 2.2.4 Evaluation metrics

This study employed a cost-utility analysis (CUA) framework to evaluate the economic performance of cervical cancer prevention strategies. The primary outcome measure was the ICER, which is widely used by healthcare decision-makers to determine whether an intervention provides sufficient value for its cost. Costs: included expenditures related to HPV vaccination, screening, and treatment. Benefits: represented the cost savings achieved by preventing cervical cancer cases through vaccination or screening. Effectiveness: measured as the number of cervical cancer cases and deaths averted via vaccination or screening.

The ICER was calculated as the difference in cost between an intervention strategy (a) and a comparator strategy (b), divided by the difference in effectiveness, as shown in Equation (1):


(1) $$\text{ICER}=\frac{C_{B}-C_{A}}{E_{B}-E_{A}}=\frac{\mathrm{\Delta C}}{\mathrm{\Delta E}}$$

An intervention was considered highly cost-effective if its ICER fell below the per capita gross domestic product (GDP), consistent with the World Health Organization (WHO) guidelines. Both costs and utilities were discounted at an annual rate of 3%. The willingness-to-pay (WTP) threshold was set at one to three times the per capita GDP.

According to the National Bureau of Statistics of China, the per capita GDP in 2023 was 89,358 CNY (approximately USD 12,400) (15), and this value was adopted as the reference threshold in this study.

#### 2.2.5 Vaccine efficacy and screening sensitivity/specificity

Cervical cancer prevention relies on two core strategies: HPV vaccination and screening. Current evidence demonstrates that HPV vaccines offer strong protection against high-risk HPV infections and related cervical lesions. In terms of screening, cytology (TCT) and high-risk HPV DNA testing are the most widely used approaches in Hainan Province. The parameters of vaccine efficacy and screening performance are summarized in Tables 4, 5. In the Markov model, the sensitivity and specificity of screening tests were applied to simulate test outcomes in each screening round:

Sensitivity: the proportion of true positives correctly identified.
Specificity: the proportion of true negatives correctly identified. False negatives were assumed to progress according to the natural history of HPV infection and cervical cancer. False positives generated additional diagnostic costs without health benefits. These modeled outcomes influenced both disease progression and the calculation of costs and QALYs.

**Table 4:** HPV vaccine parameters.

Vaccine type	Outcome	Population	Efficacy (%)	Range (%)	Reference
Bivalent	Cervical cancer	Females aged 15–25	91.7	(82.4, 96.7)	FDA (33)
	CIN1/2/3	Females aged 15–25	91.7	(82.4, 96.7)	FDA (33)
Quadrivalent	Cervical cancer	Females aged 16–26	94.8	(92.6, 98.1)	Garland SM et al. (34)
	Genital warts	Females aged 16–26	96	(87.3, 99.7)	Garland SM et al. (34)
	CIN1/2/3	Females aged 16–24	98.2	(92.1, 99.8)	Garland SM et al. (34)
9-valent	Cervical cancer	Females aged 16–45	96	(92.3, 98.2)	FDA (33)
	Genital warts	Females aged 16–45	99	(96.2, 99.9)	FDA (33)
	CIN1/2/3	Females aged 16–26	96.7	(80.9, 99.8)	Joura EA et al. (35)

**Table 5:** Screening test parameters.

Parameter	Base-case	Range	Distribution	Reference
HPV Testing				
Sensitivity	0.90	0.810–0.910	Normal	Chen (36)
Specificity	0.45	0.410–0.460		
TCT Screening				
Sensitivity	0.75	0.713–0.788		

#### 2.2.6 Sensitivity analysis

Both one-way sensitivity analysis (OSA) and probabilistic sensitivity analysis (PSA) were conducted to assess the robustness of the decision tree–Markov model and to identify key drivers of uncertainty. Sensitivity analyses determine which parameters exert the greatest influence on model outcomes and clarify how parameter variation affects the reliability of results. Parameters with negligible impact were excluded to streamline the model.

The results of the OSA were presented using a tornado diagram, which highlights the relative influence of each parameter. Sensitivity analyses are essential in economic evaluations, as variations in certain parameters may warrant reconsideration of intervention strategies (16). In this study, highly sensitive parameters caused substantial changes in model outcomes, whereas parameters with limited influence confirmed the model’s overall stability and reliability for informing decision-making.

#### 2.2.7 Model validation

A cervical cancer intervention model was developed to simulate disease progression under a no-intervention scenario. The model-predicted age-specific incidence and mortality rates were validated against data reported in the China Health Statistics Yearbook.

**Figure 3:**
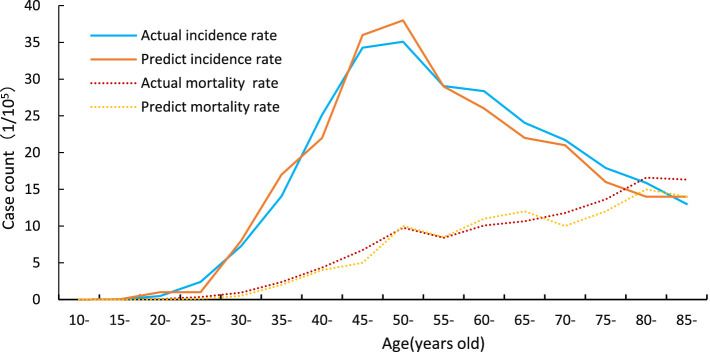
Validation of the Markov model. Calibration and validation of the cervical cancer intervention model. The two solid lines represent the observed incidence of cervical cancer and the incidence predicted by the calibrated model. The two dashed lines correspond to the actual and model-predicted cervical cancer mortality rates. The presence of two curves for both incidence and mortality reflects the comparison between observed epidemiological data and model projections under the no-intervention scenario.

The two solid lines represent the observed incidence of cervical cancer and the incidence predicted by the calibrated model. The two dashed lines correspond to the actual and model-predicted cervical cancer mortality rates. As shown in Figure 3, cervical cancer incidence begins to increase at approximately age 30, peaks around age 50, and gradually declines with fluctuations thereafter. In contrast, mortality follows an upward trend with age, with a notably higher rate observed among women aged ≧75 years. These results indicate that the calibrated model provides a reasonable approximation of the natural history of cervical cancer in China.

## 3 Results

### 3.1 Cost-effectiveness analysis of cervical cancer screening strategies

**Figure 4:**
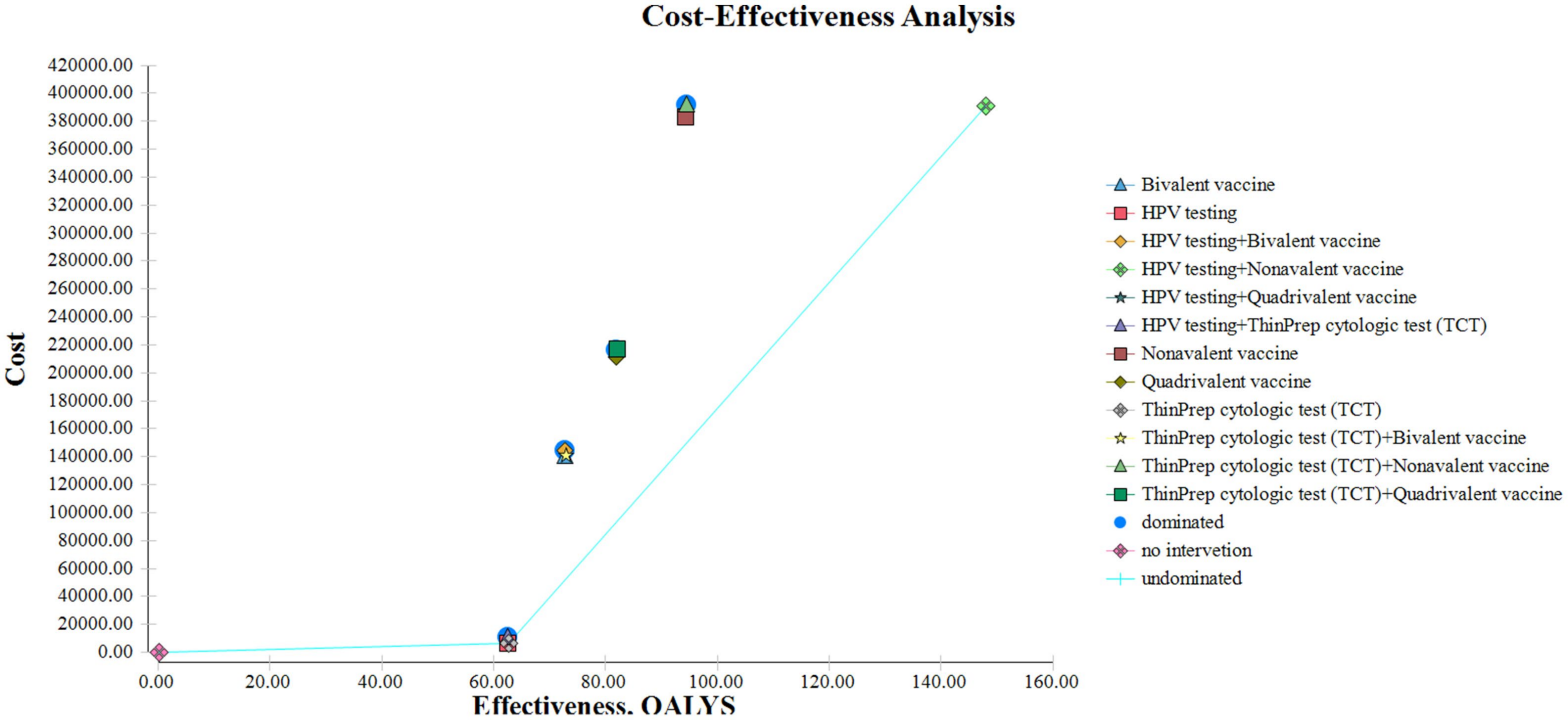
Cost-effectiveness plane of all cervical cancer prevention strategies.

A decision tree–Markov model was used to retrospectively analyze cervical cancer prevention strategies. Thirteen strategies were evaluated, including no intervention, screening alone, and combinations of screening with HPV vaccination. Cost-effectiveness was assessed under the defined WTP threshold using ICERs to compare the relative economic value of each strategy (Figure 4).

### 3.2 Cost-effectiveness analysis results

A cost-utility analysis was conducted to estimate total costs, QALYs, and ICERs for each strategy (Table 6).

**Table 6:** Cost-effectiveness analysis of different cervical cancer prevention strategies.

Strategy	Total cost	QALYS	ICER
Bivalent vaccine	14,0072.71	72.78	13,333.65
Quadrivalent vaccine	21,0955.67	81.96	7,682.12
Nonavalent vaccine	38,3076.21	94.28	13,612.24
HPV	63,2078.73	62.56	983.87
TCT	63,5192.62	62.78	14,455.05
HPV + TCT	67,4301.31	62.52	16,215.60
HPV plus bivalent vaccine	14,4765.48	72.69	19,854.891
TCT plus Bivalent vaccine	14,1147.99	72.87	12,595.655
HPV plus Quadrivalent vaccine	21,6361.55	81.92	157,540.20
TCT plus Quadrivalent vaccine	21,7321.32	82.10	42,796.441
HPV plus Nonavalent vaccine	39,0579.83	148.04	139.58
TCT plus Nonavalent vaccine	39,1613.84	94.53	193,240.60
No intervention	184,471.49	0	0

HPV screening alone provided the most favorable cost-effectiveness, with 62.56 QALYs and an ICER of ¥983.87/QALY. This approach demonstrated high efficiency and may be preferred in resource-constrained settings. In contrast, TCT screening produced slightly greater health benefits (62.78 QALYs) but at a much higher ICER (¥14,455.05/QALY), indicating limited economic value. HPV screening combined with 9-valent vaccination achieved the greatest health gains (148.04 QALYs), with a low ICER of ¥139.58/QALY despite the highest total cost (¥390,579.83). This strategy may be most appropriate in well-resourced healthcare systems.

### 3.3 Sensitivity analysis

**Figure 5:**
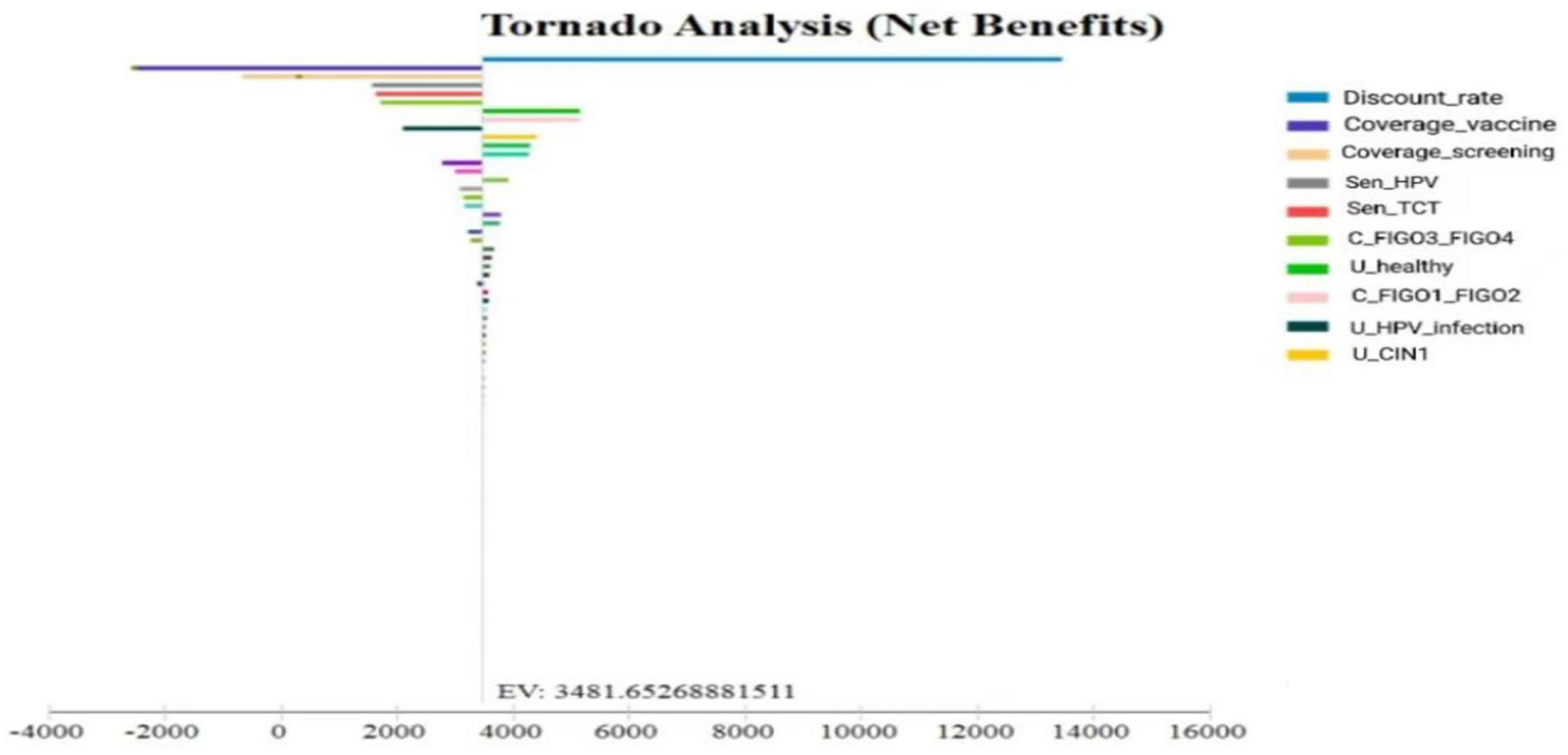
Tornado diagram of one-way sensitivity analysis.

One-way sensitivity analyses were performed on key parameters, including screening and vaccination coverage, vaccine and treatment costs, and the discount rate, using baseline values and plausible ranges. Coverage rates varied between 70 and 90%, vaccine and treatment costs varied by ±20%, and the discount rate ranged from 1 to 5%. Results were visualized using a tornado diagram (Figure 5). Unlike conventional single-bar displays, this diagram shows separate lower- and upper-bound estimates, allowing clearer interpretation of asymmetric effects.

The analysis identified vaccination uptake, test sensitivity, and the discount rate as the most influential variables. However, no parameter variation caused ICERs to exceed one-time per capita GDP or altered the ranking of the optimal strategy, supporting the robustness of the results.

**Figure 6:**
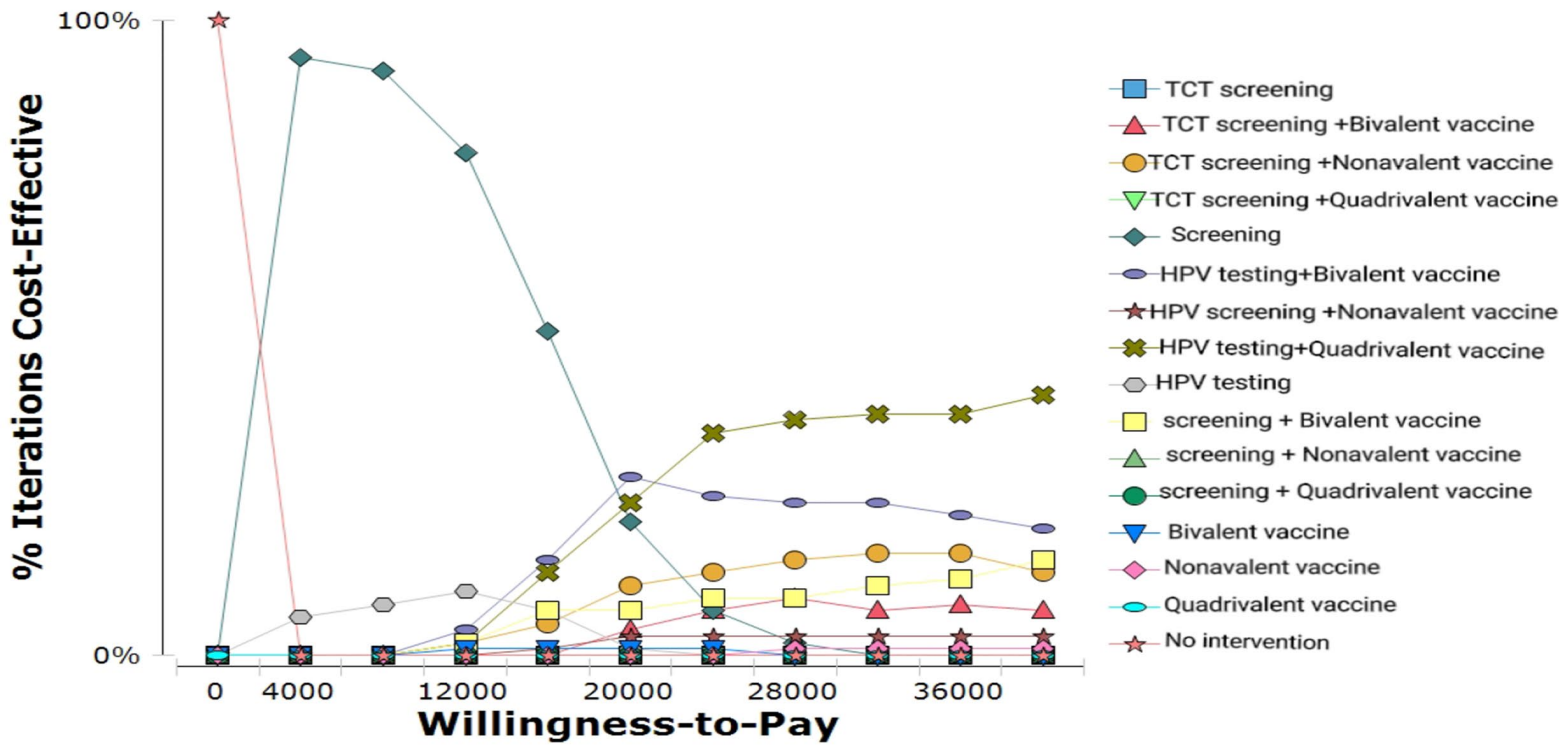
Cost-effectiveness acceptability curve.

Probabilistic sensitivity analysis (PSA) results for different strategies are shown in Figure 6. The cost-effectiveness acceptability curve (CEAC) demonstrated that:

At lower WTP levels, HPV screening alone was cost-effective in nearly 100% of simulations.

As WTP increased, the combination of HPV screening and 9-valent vaccination became progressively more cost-effective.

When the WTP exceeded ~¥20,000, this combined strategy had the highest probability of being cost-effective, establishing it as the most economically favorable option.

## 4 Discussion

This study demonstrated that the combination of HPV testing and the 9-valent vaccine was the most cost-effective strategy among all cervical cancer prevention options, with an ICER well below the per capita GDP threshold. This finding is consistent with previous studies. Mo et al. (17) conducted a simulation study in China and reported that HPV9 vaccination combined with screening provided the greatest benefits in terms of QALYs gained and ICER reduction, making it highly suitable for nationwide implementation. Similarly, Zou et al. (18) concluded that HPV testing every 5 years combined with vaccination was among the most cost-effective approaches across multiple scenarios in China.

The consistency of these results can be explained by several factors. First, HPV testing has higher sensitivity than TCT in detecting high-risk infections, enabling earlier diagnosis and intervention. Second, longer screening intervals (e.g., every 5 years) reduce the number of lifetime screening visits and associated cumulative costs. Third, the 9-valent vaccine provides broader protection against high-risk HPV serotypes, and when combined with sensitive screening, it maximizes preventive benefits while improving overall economic efficiency. Together, these factors support the robustness of our model projections.

Our sensitivity analysis further indicated that the discount rate, vaccination coverage, and screening coverage were the most influential parameters affecting ICER estimates. This aligns with the findings of Sroczynski et al. (19)in Germany, who demonstrated that variations in the discount rate had a major impact on ICER values, with lower discounting substantially improving cost-effectiveness. Similarly, Obradovic et al. (20) in Slovenia identified vaccination coverage and discounting assumptions as key drivers of cost-effectiveness outcomes.

This consistency is likely because these parameters directly influence both long-term health outcomes and total intervention costs, which together determine ICER values. Thus, across different healthcare contexts, variations in discounting assumptions and coverage rates tend to exert substantial influence on cost-effectiveness results.

Nevertheless, some studies have reported different sensitivity rankings. For example, Ekwunife and Lhachimi (21) found that in Nigeria, the unit cost of HPV vaccines had a greater effect on ICER than discount rate or screening coverage. This discrepancy may be attributed to structural differences in healthcare systems. In resource-constrained settings such as Nigeria, vaccine expenditures represent a larger proportion of total intervention costs, making price variations a dominant determinant of cost-effectiveness.

The major limitation of our study is that utility values were derived from European populations rather than Chinese-specific data. Cultural preferences, health perceptions, and disease burden vary across regions, which may result in inaccuracies in health-related quality-of-life measurements. Consequently, this substitution could bias QALY estimation and ICER outcomes, potentially under- or overestimating the true cost-effectiveness of HPV vaccination in China. Although the use of European data was unavoidable due to the lack of domestic utility values, the findings should be interpreted with caution. Future research should aim to generate China-specific utility data to improve the precision and policy relevance of cost-effectiveness evaluations.

## 5 Conclusion

The combination of 9-valent HPV vaccination and HPV screening represents the most effective and cost-efficient strategy for cervical cancer prevention in the province. By accounting for variations in vaccination and screening coverage, the Markov model provided a more comprehensive evaluation of disease incidence and progression within the existing preventive framework.

## Data Availability

The datasets presented in this study can be found in online repositories. The names of the repository/repositories and accession number(s) can be found in the article/supplementary material.
